# Contingency management interventions for substance use and addictive behaviours: Review of the United Kingdom evidence base

**DOI:** 10.1111/add.70240

**Published:** 2025-11-16

**Authors:** Carol‐Ann Getty, Tricia McQuarrie, Eileen Brobbin

**Affiliations:** ^1^ National Addiction Centre, Institute of Psychiatry, Psychology and Neuroscience King's College London London UK; ^2^ Department of Mental Health and Social Work, Faculty of Health, Social Care and Education Middlesex University London UK

**Keywords:** addictive behaviours, contingency management, evidence base, implementation science, scoping review, substance use

## Abstract

**Background and aims:**

Substance use and other addictive behaviours including gambling remain major public health concerns in the UK. Despite the effectiveness of substance use treatment approaches, treatment adherence and success rates remain low. Contingency Management (CM), a behavioural intervention using positive reinforcement, is a promising approach to enhance clinical outcomes; however, its implementation in UK settings remains limited. This scoping review aimed to explore how CM has been adopted to improve outcomes for substance use and addictive behaviours in the UK, addressing the gap between international evidence and UK‐specific healthcare needs.

**Methods:**

A systematic search of databases (Embase, MEDLINE, PsycArticles and PsycInfo) for UK‐based CM studies published before March 2025 was undertaken. The review adhered to PRISMA‐ScR guidelines, and the protocol was prospectively registered on the Open Science Framework. Eligible studies were peer‐reviewed, full‐text articles, reporting the exploration of CM interventions to improve outcomes for substance use and other addictive behaviours within UK settings. Screening and data extraction were independently conducted using Covidence. A narrative synthesis explored study outcomes including effectiveness, feasibility and acceptability. Using the Context and Implementation of Complex Interventions (CICI) framework, key contextual factors influencing CM implementation in the UK across setting, socio‐cultural, political and ethical domains were explored.

**Results:**

A total of 208 articles were identified, with 36 full texts reviewed and 29 eligible for inclusion. Nine studies assessed effectiveness, six included economic evaluations, six assessed feasibility and 14 assessed acceptability. Clinical effectiveness was supported in most studies, particularly for promoting abstinence and medication adherence. Feasibility concerns included resource limitations, training and recruitment challenges. CM was generally well‐accepted by service users and professionals, and digital approaches showed promise with high adherence and accuracy. Barriers and facilitators to CM implementation operating at micro, meso and macro levels are presented.

**Conclusion:**

This scoping review of studies implementing Contingency Management (CM) in UK addiction treatment highlights several barriers to CM adoption, including resource limitations, concerns about its impact on therapeutic relationships and ethical issues regarding manipulation and fairness. It also points to the need for adapting CM protocols to fit UK treatment philosophies, particularly aligning with harm reduction approaches. CM's success depends on multi‐level support, including policy, training and integration with existing systems. Recommendations include strengthening research on CM's long‐term impact, ensuring fidelity to core principles, and investing in digital tools to reduce administrative burden.

## INTRODUCTION

Addictive behaviours including alcohol, tobacco, illicit drug use and gambling, pose significant public health challenges, contributing to a wide range of health, social and economic issues. Between 2022 and 2023, 9.5% of United Kingdom (UK) adults reported having used a drug in the last 12 months, with 2.3% reporting to do so frequently [1]. Cannabis has consistently been the most used drug in England and Wales (7.6%), followed by powder cocaine (2.4%), nitrous oxide (1.3%), ecstasy (1.1%) and hallucinogens (1%). Opioids, although less prevalent, account for significant health and social harms and were implicated in 46% of the 4907 drug‐related deaths registered in England and Wales in 2022 [2]. The rate of drug‐poisoning deaths has increased year‐on‐year and is now 81.5% higher than in 2012. Moreover, alcohol consumption among UK adults was found to be as high as 56% in 2022, with an estimated 608 416 adults with alcohol dependence [3]. Tobacco use, although prevalence is declining (12.9% in 2022), remains the leading cause of preventable deaths in the United Kingdom [4]. Moreover, there are an estimated 1.6 million adults who gamble who may benefit from treatment or support for harmful gambling in England [5].

Between 2022 and 2023, 290 635 adults were in contact with drug and alcohol services, while 137 749 entered treatment [6]. Nearly half (48%) of those were in treatment for opiate use. Of those accessing treatment, 99% received a psychosocial intervention and 53% received at least one pharmacological intervention. Comprehensive treatment necessitates psychosocial interventions to address the intertwined psychological and social issues associated with substance use disorder (SUD). However, many do not benefit from current treatment approaches. Of the 127 385 individuals who exited drug and alcohol treatment between 2022 and 2023, less than half (46%) did so successfully, 1 while as many as 37% dropped out or left without completing treatment [6].

The effectiveness of substance use treatment approaches is substantially hampered by poor adherence. Adherence rates vary by SUD and treatment type, with opiate users having the lowest successful completion rate (23%), followed by non‐opiate drug and alcohol users (49%), non‐opiate drug users (51%) and alcohol users (58%) [6]. Adherence to tobacco‐dependence treatments was found to be as low as 25% to 30% [7, 8]. Attendance at outpatient appointments for drug and alcohol services also sees high non‐compliance rates, with between 25.3% and 36.9% of new patient appointments missed annually in the United Kingdom [9].

Behavioural interventions like contingency management (CM) are among the most promising strategies for improving SUD treatment compliance [10]. CM involves the application of positive reinforcement (e.g. monetary incentives) contingent on evidence of positive behaviour change [11]. CM is based on the theoretical principles of operant conditioning and is among the most efficacious psychosocial interventions in promoting abstinence from smoking, alcohol and illicit drugs [12, 13, 14]. The integration of CM in UK substance use treatment services is recommended by the UK National Institute for Health and Care Excellence (NICE) [15] and the ‘Orange Book’ [16] to promote therapeutic progress and positive treatment. In 2023, the Office for Health Improvement and Disparities (OHID) announced a national roll‐out of the ‘Smoke‐Free Pregnancy Incentive Scheme’, applying CM principles to support perinatal smoking cessation [17].

Despite NICE's recommendations, the evidence base is largely dominated by the United States (US), and few studies have attempted to integrate CM into a UK‐based clinical setting. Another challenge is understanding service users' and stakeholders' attitudes toward CM, because these are likely to be influential in directing future adoptions. Because of significant differences between the United Kingdom and United States healthcare systems that influence how treatments are implemented, monitored and accessed by patients, investigating the effectiveness and acceptability of CM within the United Kingdom is essential. Understanding the cultural and social nuances that affect patient behaviour and treatment adherence in the United Kingdom can help tailor CM approaches to maximise their impact on treatment outcomes. Although the field of CM benefits from an array of published systematic reviews and meta‐analyses that guide and inform researchers' and clinicians' understanding of these interventions, they are limited in their ability to provide an overview of evidence at a local level. This is particularly problematic for UK‐based healthcare professionals and practitioners who seek to understand how CM could be used to enhance treatment outcomes within their services.

The aim of this scoping review is to synthesise the existing literature on the use of CM interventions for substance use and addictive behaviours in the United Kingdom; evaluate the effectiveness, feasibility and acceptability of CM; and identify key barriers and facilitators to implementation within UK clinical settings to inform future research, policy and practice.

## METHODS

A scoping review was undertaken to comprehensively map the diverse and heterogeneous literature on CM for substance use and addictive behaviours in the United Kingdom, allowing inclusion of varied study designs and contextual factors beyond effectiveness, given the exploratory nature and limited evidence base in this area. This scoping review was conducted in accordance with Arksey and O'Malley's five‐step framework: [18] (1) identifying the research question; (2) identifying relevant studies; (3) study selection; (4) charting the data; and (5) collating, summarising and reporting results. Reporting of this scoping review has been guided by the Reporting Items for Systematic reviews and Meta‐Analyses extension for Scoping Reviews (PRISMA‐ScR) Checklist [19]. Covidence systematic review management software [20] was used to conduct the review efficiently.

### Protocol and registration

This review was registered prospectively on the Open Science Framework (OSF) registry (https://doi.org/10.17605/OSF.IO/8ZAN3) [21].

### Eligibility criteria

All UK‐based studies exploring the effectiveness, feasibility or acceptability of CM to improve treatment‐related outcomes in the United Kingdom will be included. This includes non‐interventional qualitative explorations. The inclusion criteria are full‐text, original, published studies (no time restrictions), peer‐reviewed and published in English. The rationale for these criteria was that English is the main scientific language, and peer‐reviewed articles offer a higher guarantee of quality.

### Information sources

Using the OVID platform, databases searched were: Embase, Ovid MEDLINE(R) ALL, APA PsycArticles and APA PsycInfo. Additional searches included: Registered Controlled Trial Registers and pre‐prints. The searches were supplemented by cross‐checking the reference list of relevant and key publications. Related systematic reviews were consulted to identify any additional studies.

### Search strategy

The search strategy included keywords related to substance use disorder, the intervention and context of interest combined with Boolean operators AND and OR. The final search was conducted on 20th March 2025. Search terms included: ‘contingency management’, ‘United Kingdom,’ UK, addiction, drug, gambling, ‘addictive behaviour,’ substance, alcohol, England, Wales, ‘Northern Ireland,’ Scotland, disorder, opioid, cocaine and nicotine. A hand search for eligible articles not captured by our search was also carried out. A detailed search strategy can be found in the OSF registry [21].

### Selection of sources of evidence

After the search strategy was carried out, all titles and abstracts were screened independently by two reviewers (C.A.G., T.M.) to identify all studies that potentially met the inclusion criteria described. Any doubts were discussed and screened by the third reviewer (E.B.). From the list of potential eligible studies, the full texts were retrieved and assessed for eligibility by two reviewers (C.A.G., E.B.). Any doubts were discussed and screened by a third reviewer (T.M.).

### Data charting process

A data extraction form was created and pilot tested with the first three included studies and refined as necessary. Data extraction was evenly shared among the three reviewers, with a subset of entries cross‐checked (20% or two, whichever is greater). Authors of studies were contacted by reviewers to clarify existing data, to request missing data or additional data.

### Data items

Data extracted included study characteristics, participant characteristics, overview of intervention, comparator (if appropriate) and outcome data/findings.

### Synthesis of results

A narrative synthesis of the findings from the included studies is provided. This is structured around study outcomes, including effectiveness, feasibility and acceptability and the key contextual domains impacting CM implementation according to the ‘Context and Implementation of Complex Interventions’ (CICI) framework [22].

## RESULTS

### Study selection

**FIGURE 1:**
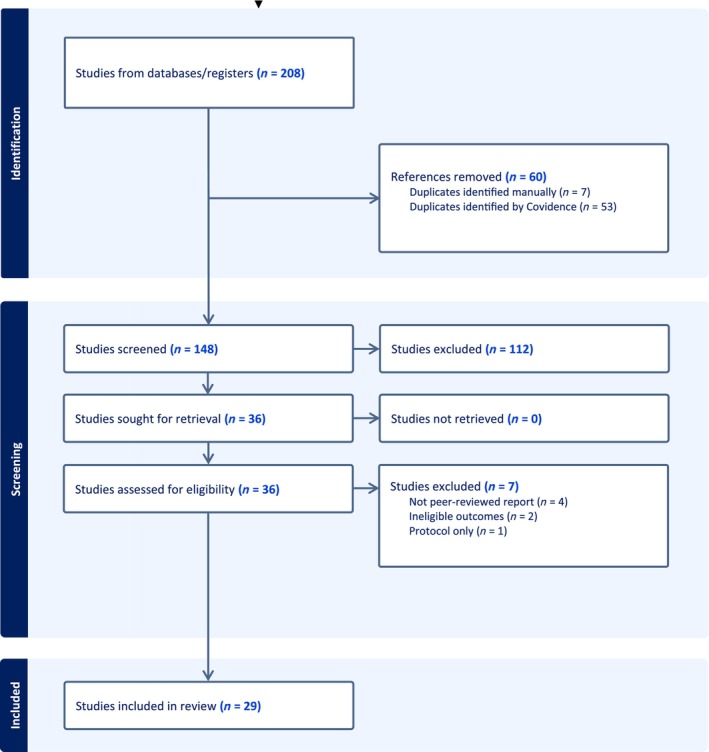
Preferred Reporting Items for Systematic Reviews and Meta‐Analyses (PRISMA) flow diagram.

A total of 208 articles were identified through searches. Following deduplication, 148 titles and abstracts were screened. Of those, 112 articles were excluded. The remaining 36 full texts were reviewed. Seven articles were excluded: two reported ineligible outcomes, while five were not full‐text peer‐reviewed articles. Twenty‐nine studies were eligible for inclusion. See Figure 1 for the PRISMA flow diagram.

### Characteristics of included studies

Included studies are categorised according to the primary outcomes reported, including: (1) effectiveness of CM including clinical outcomes such as abstinence, attendance, medication adherence, vaccination uptake, treatment engagement and cost‐effectiveness (Table 1); (2) feasibility of implementing CM or conducting CM confirmatory trials (Table 2); and (3) acceptability among treatment providers, staff or service users (Table 3).

**TABLE 1:** Characteristics of studies exploring effectiveness.

Study author(s) (y)	Design	Population	Sample size (baseline)	Comparison	Length of follow‐up	Treatment duration	Target behaviour	Overview of CM	Outcomes
Tappin *et al*. [23] (2015)	Single blind RCT	Pregnant smokers	612	Standard smoking services	Up to late pregnancy (34–38 weeks); 6‐month postnatal for confirmed quitters	Up to 38 weeks	Engagement with current stop smoking services or to stop smoking, or both, during pregnancy	Up to £400 in vouchers contingent on verified quit milestones	23% in CM arm vs. 9% in control arm stopped smoking; relative risk of not smoking at the end of pregnancy was 2.63 (95% CI = 1.73–4.01), *P* < 0.001
Boyd *et al*. [24] (2015)^a^	Economic evaluation	^a^Data from Tappin *et al*. [23] (2015)	^a^Data from Tappin *et al*. [23] (2015)	^a^Data from Tappin *et al*. [23] (2015)	^a^Data from Tappin *et al*. [23] (2015)	^a^Data from Tappin *et al*. [23] (2015)	^a^Data from Tappin *et al*. [23] (2015)	^a^Data from Tappin *et al*. [23] (2015)	Incremental cost per quitter at 34–38 weeks pregnant was £1127; lifetime model estimated an incremental cost of £17 and a gain of 0.04 QALYs, resulting in an ICER of £482/QALY
Tappin *et al*. [25] (2022)	Pragmatic, multicentre, single blinded RCT	Pregnant smokers	944	Standard smoking services	Up to late pregnancy (34–38 weeks); 6‐month postnatal for confirmed quitters	Up to 38 weeks	Engagement with current stop smoking services or to stop smoking, or both, during pregnancy	Up to £400 in vouchers contingent on verified quit milestones	27% in CM arm vs. 12% in control arm stopped smoking [adjusted OR = 2.78 (1.94–3.97) *P* < 0.001]
Sheridan Rains *et al*. [26] (2019)	Pragmatic multi‐centre RCT	EIP service users	551	CM plus psycho‐educational intervention vs. psycho‐educational intervention only.	18 months	12 weeks	Cannabis reduction	Escalating vouchers for cannabis‐negative UDS	No statistically significant difference in time to acute psychiatric care (HR = 1.03, 95% CI = 0.76–1.40) between groups
Sheridan Rains *et al*. [26] (2019)^a^	Economic evaluation	^a^Data from Sheridan Rains *et al*. [26] (2019)	^a^Data from Sheridan Rains *et al*. [26] (2019)	^a^Data from Sheridan Rains *et al*. [26] (2019)	^a^Data from Sheridan Rains *et al*. [26] (2019)	^a^Data from Sheridan Rains *et al*. [26] (2019)	^a^Data from Sheridan Rains *et al*. [26] (2019)	^a^Data from Sheridan Rains *et al*. [26] (2019)	81% likelihood that the intervention was cost‐effective, mainly because of higher inpatient costs in the control group; however, the cost difference was not statistically significant
Metrebian *et al*. [27] (2021)	Pragmatic cluster RCT	Service users receiving OAT	552	CM for abstinence, CM for attendance, no CM	24 weeks	12 weeks	Heroin abstinence	Fixed vouchers for attendance at weekly appointments or opiate‐negative UDS	CM attendance significantly improved heroin abstinence over TAU in weeks 9–12 (OR = 2.1; *P* = 0.030), while CM abstinence showed no significant differences from TAU or CM attendance; effects were not sustained at weeks 21–24
Shearer *et al*. [28] (2023)^a^	Economic evaluation	^a^Data from Metrebian *et al*. [27] (2021)	^a^Data from Metrebian *et al*. [27] (2021)	^a^Data from Metrebian *et al*. [27] (2021)	^a^Data from Metrebian *et al*. [27] (2021)	^a^Data from Metrebian *et al*. [27] (2021)	^a^Data from Metrebian *et al*. [27] (2021)	^a^Data from Metrebian *et al*. [27] (2021)	Incremental cost‐effectiveness ratios were £10 167 per additional heroin‐free urine for CM abstinence and £3562 for CM attendance with low cost‐effectiveness probabilities of 3.5% and 36%, respectively; at 12 weeks, CM Attendance dominated TAU with an 88.4% probability of cost‐effectiveness, while CM abstinence remained low at 8.6%
Marsden *et al*. [29] (2019)	Pragmatic, parallel‐group, open‐label, RCT	Service users receiving OAT	273	CM for attendance, recovery activities or abstinence vs. TAU	18 weeks	12 weeks	Clinic attendance, recovery activities, or drug abstinence	Vouchers for treatment‐related behaviours as part of toolbox of psychological interventions	At 18 weeks, 16% in the psychosocial intervention group were treatment responders compared with 7% in the control group [adjusted log odds = 1·20 (95% CI = 0.01–2.37); *P* = 0·048]
Marsden *et al*. [29] (2019)^a^	Economic evaluation	^a^Data from Marsden *et al*. [29] (2019)	^a^Data from Marsden *et al*. [29] (2019)	^a^Data from Marsden *et al*. [29] (2019)	^a^Data from Marsden *et al*. [29] (2019)	^a^Data from Marsden *et al*. [29] (2019)	^a^Data from Marsden *et al*. [29] (2019)	^a^Data from Marsden *et al*. [29] (2019)	The psychosocial intervention was more likely to be cost‐effective, with a 47%–87% probability of cost‐effectiveness at willingness‐to‐pay thresholds of £0–1000 per unit improvement in treatment response; it also yielded higher QALYs (mean difference 0.048; *P* = 0.004), with 60% and 67% probabilities of cost‐effectiveness at £20 000 and £30 000 per QALY thresholds, respectively
Moss *et al*. [30] (2020)	Cohort study, retrospective analysis of outcome data following implementation of capital card	Service users in SUD treatment	1545	CM (capital card) vs. TAU	24 months	Not stated	Treatment activities including attendance and engagement with harm reduction interventions	Points‐based system for engaging with treatment	Clients with a capital card were 1.5 times more likely to successfully complete treatment than those who did not have one (OR = 1.507, 95% CI = 1.194–1.902)
Smith *et al*. [31] (2025)	Cohort study, retrospective analysis of outcome data following implementation of WAND initiative	Service users who inject drugs and participating in WAND harm reduction initiative	831	Two groups engaging with WAND those who engaged once and those who re‐engaged on more than one occasion	12 months	12 months	Completion of WAND harm reduction interventions	Voucher on completion of all four interventions and at each 3 month re‐engagement	40% re‐engagement in WAND
Weaver *et al*. [32] (2014)	Cluster RCT	Service users in SUD treatment and currently (or at risk) of injecting	210	Hep b vaccination completion plus fixed‐CM or escalating CM or no CM	12 weeks	4 weeks	Completion of hep b vaccination	Fixed CM Vaccination with three £10 vouchers or escalating CM vaccination with three vouchers £5, £10, £15	45% (fixed CM), 49% (escalating CM), 9% (TAU) of participants completed hep b vaccinations (OR = 12·1 and NNT of 2·78 for fixed CM and OR = 13·9 for escalating CM and NNT of 2·48) with sensitivity analyses
Rafia *et al*. [33] (2016)^a^	Economic evaluation	Data from Weaver *et al*. [32] (2014)	Data from Weaver *et al*. [32] (2014)	Data from Weaver *et al*. [32] (2014)	Data from Weaver *et al*. [32] (2014)	Data from Weaver *et al*. [32] (2014)	Data from Weaver *et al*. [32] (2014)	Data from Weaver *et al*. [32] (2014)	The incremental life‐time healthcare cost of CM vs. usual care was £21.86 (95% CI = −£12.20 to 39.86) per person; for 1000 people, 19 hep b infections (95% CI = 8–30) were avoided; the probabilistic incremental cost per QALY gained was £6738 (95% CI = £6297–7172), with an 89% probability of cost‐effectiveness at £20 000 per QALY (97.6% at £30 000)
Donoghue *et al*. [34] (2023)	Multi‐centre, three‐arm, parallel‐group, randomised controlled clinical trial	Service users with a diagnosis of alcohol dependence	739	SS, SS + MM, SS + MM + CM	6 months	6 months	Attendance at MM sessions	Vouchers delivered to encourage attendance at pharmacist‐led telephone support for acamprosate adherence; calls were weekly for 6 weeks, then fortnightly for 6 weeks, then monthly for 3 months	The primary outcome was self‐reported medication adherence over 6 months. SS + MM + CM significantly improved adherence to acamprosate compared to SS alone (mean difference 10.6%; 95% CI = 1.6%–19.6%); no significant differences were found between SS + MM and SS alone (3.1%; 95% CI = −6.5% to 12.8%) or SS + MM and SS + MM + CM (7.9%; 95% CI = −2.8% to 18.7%)
Donoghue *et al*. [34] (2023)^a^	Economic evaluation	Data from Donoghue *et al*. [34] (2023)	Data from Donoghue *et al*. [34] (2023)	Data from Donoghue *et al*. [34] (2023)	Data from Donoghue *et al*. [34] (2023)	Data from Donoghue *et al*. [34] (2023)	Data from Donoghue *et al*. [34] (2023)	Data from Donoghue *et al*. [34] (2023)	At 6 months SS and MM with CM was cost‐effective compared to SS alone, with small QALY gains at a lower cost Adjunctive MM showed no cost‐effectiveness compared to SS

Abbreviations: CM, contingency management; EIP, early intervention in psychosis; hep b, hepatitis B; OAT, opioid agonist treatment; QALY, quality‐adjusted life year; SS, standard support; SS + MM, standard support with medication management; SS + MM + CM, standard support and medication management with contingency management; SUD, substance use disorder; TAU, treatment‐as‐usual; WAND, wound care, assessment of injecting risk, naloxone, dry blood spot test.

**TABLE 2:** Characteristics of studies exploring feasibility.

Study author(s) (y)	Design	Population	Sample size (baseline)	Comparison	Length of follow‐up	Treatment duration	Target behaviour	Overview of CM	Outcomes
Seel *et al*. [35] (2024)	Case study design	Individuals with gambling disorder	1	None	8 weeks	8 weeks	Attendance in weekly research meetings (weeks 1–2); attendance, abstinence and recovery goals in week 3–8	Points‐based system for attendance and abstinence	CM procedures were acceptable to the client and can be practicably applied as an adjunct to other therapeutic provision for gambling addiction
Metrebian *et al*. [36] (2021)	Three‐arm cluster randomised feasibility trial	Service users receiving OAT	10	CM plus OAT, reminders plus OAT, OAT only	12 weeks	12 weeks	Supervised consumption of methadone	Financial incentive with bonus for daily adherence of supervised OAT	Telephone system deemed reliable (96% consistency in recording medication adherence by self‐login vs. pharmacy records); poor recruitment rate (173 individuals were screened and 10 enrolled)
Curran *et al*. [37] (2023)	Qualitative exploration	Staff including EIS managers and multidisciplinary staff	47	None	N/A	N/A	N/A	N/A	Concerns around resource constraints, wider systemic processes and competing priorities relating to clinical guidelines and commissioning
Ainscough *et al*. [38] (2021)	Two‐armed, randomised, pilot/feasibility study	Service users receiving OAT who wish to quit smoking	19	Abstinence vs. attendance	6 weeks	5 weeks	Smoking cessation	Escalating with reset reinforcers for abstinence	Smoking clinic was poorly attended; poor retention following randomisation (particularly to abstinence condition); stricter breath CO required
Brobbin *et al*. [39] (2025)	A pilot randomised feasibility study	Alcohol dependent and in treatment in NHS services	32	No/low alcohol consumption vs. control	2 weeks	2 weeks	No/low alcohol consumption (measured by transdermal alcohol sensor)	Fixed plus bonuses	32 of a target of 30 recruited; 3 participants withdrew (2 = control, 1 = CM); 91.1% of meetings attended. BACtrack Skyn remains an accurate and acceptable tool to monitor alcohol consumption
Hemrage *etal*. [40] (2025)	A pilot randomised feasibility study	Service users with a comorbid diagnosis of AUD and ARLD	30	ILC plus CM vs. ILC only	12 weeks	12 weeks	Treatment attendance	Fixed plus bonuses	Recruitment and retention rates were 73.2% and 36.7%, respectively; all participants received CM as planned; engagement was higher in ILC + CM vs. ILC alone (67% vs. 33%); among engaged participants, alcohol intake dropped by 76% and liver outcomes improved, although no significant differences were found between groups

Abbreviations: ARLD, alcohol related liver disease; AUD, alcohol use disorder; CM, contingency management; ILC, integrated liver care; NHS, National Health Service; OAT, opioid agonist treatment.

**TABLE 3:** Characteristics of studies exploring acceptability.

Study author(s) (y)	Design	Population	Sample size (baseline)	Findings
Getty *et al*. [41] (2022)	Longitudinal qualitative interviews	Service users receiving OAT (CM recipients)	9	CM was acceptable and well received by patients and contributed to the development of enhanced and sustained adherence to their supervised methadone
Seel *et al*. [35] (2024)	Case study including qualitative interviews	Individuals receiving treatment for gambling disorder (CM recipients)	1	CM deemed acceptable and beneficial to abstinence
Curran *et al*. [37] (2023)	Qualitative focus groups and interviews	Staff including EIS managers and multidisciplinary staff (including those involved in the delivery of CM)	47	Enthusiasm for new evidence‐based interventions but concerns around the ethics of using CM for cannabis reduction and impact on therapeutic relationships
Neale *et al*. [42] (2015)	Qualitative interviews	Service users receiving OAT (CM recipients) and staff (involved in delivery of CM)	30 (20 service users; 10 staff)	Intervention delivery was influenced by patient motivation and trust, with outcomes affected by the transparency and speed of incentive delivery; unintended effects included positives (enhanced wellbeing, job satisfaction, relationships) and negatives (relapse, staff workload, clinic tensions)
Sinclair *et al*. [43] (2011)	Qualitative focus groups	Staff and service users from drug treatment services (not CM recipients)	70 (14 service users; 56 staff)	Themes include how CM was aligned to the philosophy of substance use services; the practicalities of implementation; wider ethical concerns; and how participants perceived the evidence for effectiveness
Dhital *et al*. [44] (2022)	Qualitative focus groups	Service users receiving alcohol treatment (not CM recipients)	26	Themes included concerns about support and availability of alcohol relapse prevention medication; lack of knowledge and understanding about acamprosate treatment; positive perceptions of acamprosate adherence telephone support from pharmacists; and negative perceptions of CM to support acamprosate adherence
Dorey *et al*. [45] (2022)	Qualitative interviews	Service users receiving gambling treatment (not CM recipients)	25	CM broadly supported and seen as a facilitator of extended engagement in treatment. Also concerns around CM triggering relapse among those in gambling recovery
Getty *et al*. [46] (2021)	Survey	Service users in SUD treatment (not CM recipients)	181	81% of participants were in favour of incentive programs, with more than 70% of respondents agreeing with the majority of positive belief statements; positive beliefs toward CM, with high levels of acceptability toward a range of target behaviours, incentives and the use of technology devices to remotely monitor behaviour and deliver incentives
Zolkwer *et al*. [47] (2023)	Cross‐sectional surveys	Public and students (not CM recipients)	158	CM considered beneficial in encouraging gambling cessation
McGarrigle *et al*. [48] (2023)	Survey	Gambling treatment providers (not involved in CM delivery)	111	CM for gambling deemed acceptable; willingness to use CM; common concerns involved the potential negative consequences for clients when incentives are withdrawn and the feasibility of objectively verifying gambling abstinence
Dorey *et al*. [49] (2022)	Qualitative interviews	Gambling treatment providers (not involved in CM delivery)	30	Concerns around impact on therapeutic relationship and CM triggering relapse among those in gambling recovery; acceptability varied across target behaviours and types of incentives
Metrebian *et al*. [36] (2021)	Focus groups	Service users receiving OAT (CM recipients)	10	CM participants were positive about using self‐login, the text messages and debit card; prescribers found weekly reporting, time saving and allowed closer monitoring of patients; pharmacists reported that the tablet device was easy to host
Hemrage *et al*. [50] (2024)	Longitudinal qualitative interviews	Service users receiving alcohol treatment (CM recipients)	24	Positive views toward voucher‐based CM; CM regarded as having a symbolic value and strengthening the therapeutic alliance with healthcare providers
Getty *et al*. [51] (2025)	Qualitative interviews	Addiction specialists including commissioners, policy professionals and clinicians	22	Specialists view CM as an effective addiction treatment but face challenges in its implementation, including its complexity, lack of awareness and limited resources; negative societal and media perceptions, along with political concerns, also hinder its acceptance and use

Abbreviations: CM, contingency management; OAT, opioid agonist treatment; SUD, substance use disorder.

### Participant characteristics

**FIGURE 2:**
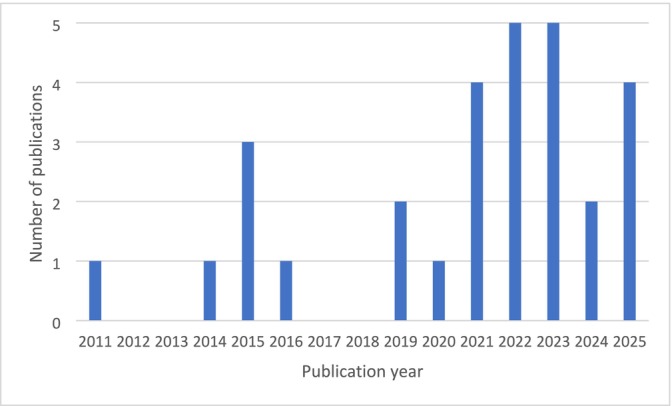
Number of studies published by year.

Of the 29 publications included in this review, only one included laypeople, while 28 were conducted within primary and secondary care facilities, specifically substance use and addiction treatment settings, mental health services, stop smoking services and community pharmacies. Publications included comprised of 6973 enrolled participants, 2 including 6648 service users, 276 staff and 158 members of the public. Service users included those receiving treatment for SUD (*n* = 2781), cannabis use disorder (*n* = 552), opioid use disorder (*n* = 874), gambling disorder (*n* = 26), alcohol use disorder (*n* = 851) and smoking cessation (*n* = 1556). The studies included were published between 2011 and 2025 (Figure 2).

### Effectiveness (clinical and cost)

Included in this review are studies that evaluated both the clinical effectiveness and cost‐effectiveness of CM interventions.

#### Clinical effectiveness

Nine studies evaluated CM in treatment populations [23, 25, 26, 27, 29, 30, 31, 32, 34]. Two encouraged heroin abstinence [27, 29], two encouraged smoking cessation [23, 25], one examined a service‐wide points system [30], one aimed to improve hepatitis B vaccination completion [32], one focused on cannabis abstinence in early psychosis [26], one targeted harm reduction engagement [31] and one addressed medication management [34].

Eight studies reported positive outcomes [23, 25, 27, 29, 30, 31, 32, 34]. CM as part of a personalised psychosocial intervention, improved abstinence, recovery activity participation and clinic attendance compared to treatment‐as‐usual (TAU) [29]. It also enhanced hepatitis B vaccination completion [32] and acamprosate adherence [34]. One study found that CM promoting appointment attendance improved heroin abstinence, but CM directly targeting abstinence did not [27]. Two studies found CM highly effective in promoting smoking cessation among pregnant smokers [23, 25]. Service‐wide CM showed higher treatment completion [30] and greater harm reduction engagement [31]. Only one study, targeting cannabis use in psychosis, reported no significant benefit [26].

#### Cost effectiveness

Six studies included economic evaluations [24, 26, 28, 29, 33, 34]. CM to boost hepatitis B vaccination and appointment attendance was cost‐effective during treatment [28, 33], although not beyond 12 weeks in the latter. CM for cannabis use showed an 81% probability of cost‐effectiveness, but the cost difference was not significant because of higher inpatient costs in the control group [26]. CM combined with psychosocial interventions yielded better outcomes at a lower cost than treatment as usual [29]. CM for smoking cessation was highly cost‐effective, with an incremental cost per quality‐adjusted life year (QALY) of £482 (reported as well below the recommended decision threshold) [24]. CM for medication adherence was also cost‐effective, offering modest QALY gains at reduced cost [34].

### Feasibility

Six studies assessed the feasibility of implementing CM [35, 36, 37, 38, 39, 40]. One study in mental health settings for cannabis reduction in psychosis identified staff concerns about limited NHS (National Health Service) resources, time, costs, training and unclear guidance [37]. Another study on CM for smoking cessation in opioid agonist treatment (OAT) populations reported low engagement and retention, and challenges verifying abstinence because of biochemical limitations [38]. A mobile CM intervention for methadone adherence showed accurate self‐verification and effective incentive delivery, but poor recruitment [36]. CM using transdermal alcohol sensors showed high recruitment, retention and attendance, with the sensors deemed accurate and acceptable [39]. A 12‐week CM trial for alcohol use disorder was feasible, with higher engagement, reduced alcohol intake and improved liver outcomes when combined with Integrated Liver Care [40]. Last, a gambling‐focused CM study faced recruitment issues, but the single case suggested potential feasibility and benefit [35].

### Acceptability

Fourteen studies explored the acceptability of CM [35, 36, 42, 43, 45, 46, 50, 51, 52]. Studies included service users undergoing treatment for SUD (*n* = 2), opioid use disorder (*n* = 3), gambling use disorder (*n* = 2), alcohol use disorder (*n* = 2), professionals (*n* = 5) and members of the public (*n* = 1). Six studies involved participants who were recipients of CM or involved in CM delivery, while eight studies explored acceptability more generally among individuals with no experience of CM.

#### Acceptability among service users

Nine studies examined service users' acceptability of CM [35, 36, 42, 43, 45, 46, 50, 51, 52]. Five involved recipients of CM, who generally reported positive experiences, viewed CM as a strong motivator that supported treatment adherence, substance use reduction and psychological well‐being [35, 36, 42, 45, 50]. CM fostered trust in providers and was seen as helpful even after incentives ended, suggesting potential for lasting behaviour change [36, 42, 50]. Digital CM interventions were well received, with participants appreciating remote incentives, structured bonuses and increased accountability.

Four studies explored acceptability among those with no experience of CM [43, 44, 45, 46], who largely supported incentives as motivational tools for treatment adherence. One study found 81% were in favour of CM, with high acceptance across behaviours and incentive types [46]. Another emphasised tailoring CM to those most in need, aligning with vertical equity principles [43]. Some concerns emerged about misuse or ethical risks, including the possibility of relapse or manipulation for financial gain [45]. One study argued that the impact of CM depends on participants' pre‐existing motivation to change, and external incentives might not always be enough for behaviour modification: those already determined to reduce or abstain benefited more [42]. Unclear information and operational inefficiencies, such as delays in receiving incentives can negatively impact the fidelity of the CM intervention and diminish the effectiveness.

#### Acceptability among professionals

Six studies examined professionals' views on CM [36, 42, 43, 48, 49, 51]. Overall, CM was seen as a useful tool to boost motivation and treatment engagement. Factors such as patient motivation, staff involvement, trust in services and treatment stability were deemed influential in CMs success [42]. Professionals noted benefits like improved job satisfaction and therapeutic relationships and showed openness to training and CM research [48, 49]. However, concerns were raised about increased workload, ethical issues and the risk of relapse after incentive withdrawal [48, 49]. Verification of target behaviours and alignment with service ethics, including fairness and autonomy, were also noted [43, 48]. Addiction specialists viewed CM as effective but cited complexity, low awareness and resource limitations as barriers [51]. Digital delivery was seen as a promising solution, although concerns about media perception and political backlash persisted. Despite this, a pragmatic stance was expressed: if CM is effective, it should be used.

#### Acceptability among lay persons

One study explored lay persons' perceptions of CM (students and the public). Within the context of voluntary self‐exclusion, gambling‐related harm reduction measures, they found positive attitudes toward the viability of CM to help those who experience problematic or harmful gambling [47]. However, participants shared concerns about CM promoting gambling or fraud‐related activities for some individuals.

### Acceptability, feasibility and contextual factors impacting implementation

Applying the CICI framework [22] to these findings can help us assess the context surrounding CM implementation and identify the barriers and facilitators to implementing CM into a complex addiction treatment system. The CICI framework consists of three key dimensions (context, implementation and setting) and was used to map out the key factors influencing CMs implementation in the United Kingdom. The framework illustrates how these elements operate at micro, meso and macro levels. Understanding the interplay between these levels and domains is essential for overcoming the barriers to the successful implementation of CM in addiction treatment services in the United Kingdom.

According to the UK literature included in this review, the key contextual domains impacting CM implementation were the setting domain (e.g. specific physical location where CM is put into practice), the socio‐cultural domain (e.g. behaviour patterns and selected ideas and values that are shared among members of a group, individuals' knowledge, beliefs and conceptions of CM), the political domain (e.g. the health care system and its accessibility including delivery of services, leadership and governance) and the ethical domain (e.g. beliefs, standards of conduct, political/media sensitivities and principles that guide the behaviour of individuals and institutions).

Micro‐level focuses on the setting domain that includes individual‐level factors such as the service users' motivations, perceptions and experiences with the CM interventions. This includes the acceptability and impact of CM on individual behaviour and psychological outcomes. Meso‐level focuses on institutional factors, such as the support or challenges within healthcare systems or organisations (e.g. the NHS). Organisational and staff‐level factors overlap and play a key role in CM implementation success, such as training, verification of behaviours and organisational support. Macro‐level focuses on system‐wide or societal influences such as national policies, economic factors and public opinions. Barriers and facilitators at this level include resource limitations such as funding, staffing, national guidelines and media coverage. Table 4 summarises the key factors at a micro, meso and macro level.

**TABLE 4:** CICI framework mapping for feasibility and acceptability of CM.

Level	Domains	Barriers	Facilitators
Micro level (refers to the level of direct action)	Setting; socio‐cultural	Resources limited staff time, high caseloads, intervention supervision, staff training Concerns about impact on therapeutic relationships Risk of CM manipulation or relapse when incentives stop Potential to undermine intrinsic motivation and patient trust	Staff involvement in CM improves effectiveness and job satisfaction Strengthens therapeutic relationships Fosters a non‐judgmental environment Positive perceptions and attitudes toward CM Openness to training and research Use of technology for CM delivery
Meso level	Ethical	Concerns about CM's alignment with substance use treatment philosophy Potential negative impact on therapeutic relationships Risk of CM manipulation or relapse when incentives stop Challenges in defining eligibility, target behaviours and incentives	Clear ethical guidelines and processes can mitigate risks Structured decision‐making can improve CM fidelity Objective verification of target behaviour Organisational support
Macro level	Political	Limited NHS and third‐sector resources High caseloads and staff shortages Training requirements and direct costs Competing priorities in clinical guidelines and funding strategies Negative media attention Political sensitivities	Strong evidence base supporting CM National guidelines recommend CM Framework for implementation

## DISCUSSION

This scoping review addressed an important gap in the literature by exploring how CM interventions have been adopted to improve outcomes for substance use and other addictive behaviours within UK settings. Findings from 29 studies addressing effectiveness, feasibility and acceptability outcomes were collated and summarised.

CM was effective in most studies, promoting abstinence from cocaine [23], smoking cessation [30, 31], increasing recovery engagement [23, 26], and improving hepatitis B vaccination rates [49]. CM was found to be effective in encouraging heroin abstinence compared with no CM only when targeted at attendance [24] and ineffective for cannabis use and psychiatric admission [27]. Economic evaluations showed CM was cost‐effective for promoting smoking cessation in pregnancy [24], vaccination uptake/completion [33], short‐term attendance [28] and when combined with psychosocial interventions [29]. CM for cannabis use showed promise, but lacked statistically significant cost differences [26].

Feasibility studies revealed implementation challenges, including limited resources, training needs and competing clinical priorities [37]. Barriers such as low engagement, retention and difficulty verifying behaviours were noted. Digital solutions showed promise with high adherence and accuracy [36, 39], although recruitment remained difficult, with one study recruiting only 6% of those screened [36]. Similar challenges were noted in other trials [35, 38].

Service users generally found CM acceptable and motivating, supporting its role in improving adherence, substance use reduction and well‐being. Remote delivery and behavioural monitoring were well received, with technology seen as beneficial [39, 41, 46]. Professionals viewed CM positively for boosting motivation, engagement and therapeutic relationships [42, 51], but raised concerns about workload, resource demands, ethical risks and alignment with service values. Barriers included low awareness, complexity and negative public perception [51]. Tailoring CM for clinical non‐responders (vertical equity) was suggested [40], although risks of misuse remained a concern [49].

The successful implementation of CM is influenced by various factors at the micro, meso and macro levels. Understanding these barriers and facilitators provides insight into how CM can be integrated effectively within existing healthcare frameworks.

### Micro‐level (direct action within clinical settings)

At the micro level, challenges include limited staff time, fears that CM may reduce intrinsic motivation or be misused by clients, and doubts about the sustainability of behaviour change once incentives end [53]. Although there is inconsistent evidence about the long‐term efficacy of CM, a recent review reported that 29% of studies examining the long‐term effects of CM reported sustained benefits even after the reinforcers had been discontinued. Notably, the longest duration of abstinence achieved during treatment strongly predicts long‐term abstinence [54]. Concerns around the longevity of behaviour change are not unique to CM, as many behaviour change interventions show limited effects beyond their active phase [55]. Gradual tapering of incentives and integration with broader recovery strategies are recommended.

Facilitators at this level include increased job satisfaction and improved therapeutic relationships [41, 42]. Openness to receiving training and engaging in CM‐based research was a critical facilitator reported [48, 49]. This is consistent with wider literature demonstrating that training workshops help increase CM‐related knowledge and correct misperceptions that may hinder adoption. Providing ongoing education, training and consultation can improve clinician understanding and support successful implementation, ultimately benefiting patient outcomes. Research confirms that staff with training or experience in CM delivery have more favourable attitudes toward CM, making adoption more likely [56, 57].

Finally, leveraging technology in CM delivery offers innovative solutions to streamline processes and reduce administrative burden as highlighted in included studies [42] and the wider field [58, 59]. It is critical that the target behaviour be monitored regularly, reinforced as immediately as possible, and objectively quantified [60]. Doing so increases the chance that each occurrence of the behaviour is reinforced and strengthens the learnt connection between abstinence and the reinforcer. Addiction specialists highlighted the potential of technology‐based approaches to facilitate this, easing workload concerns and optimising resource use [51]. A key suggestion was the development of an app to automate aspects of CM administration, simplifying implementation and reducing staff time. Integrating CM into existing client management platforms was also seen as beneficial, allowing for seamless tracking, automated notifications and enhanced reinforcement mechanisms. This technological shift was viewed as essential for making CM more accessible and sustainable within current constraints.

### Meso‐level (organisational factors)

At the meso level, interpersonal, organisational and ethical concerns shape CM implementation. Professionals expressed uncertainty about CM's compatibility with treatment models that emphasise intrinsic motivation and personal responsibility [43, 51]. These concerns often stem from misconceptions that CM undermines intrinsic motivation, which is a concept defined by an individual's desire to engage in a particular behaviour because it is personally rewarding to them and is, therefore, considered an important component of substance use and addictive behaviours. However, over time, intrinsic rewards such as improved relationships, better health and personal achievement can be established and sustain behaviour. Much of the research on the effects of reinforcers on intrinsic motivation stems from cognitive evaluation theory, which proposes that intrinsic motivation is essential for sustained behaviour change and that external reinforcers shift the locus of causality from factors internal to the individuals to factors external to the individuals and, therefore, diminish intrinsic motivation [61]. This is thought to explain why behaviour change sometimes reverts to baseline once CM is removed. However, a meta‐analysis (including high‐interest and low‐interest tasks, unlike the previous work that only included high‐interest tasks) concluded that reinforcers do not undermine intrinsic motivation but enhance it [62]. Greater understanding of the mechanisms involved in CM efficacy and its impact on motivation to change will enable more definitive conclusions to be made.

Concerns also exist around CM creating transactional therapeutic dynamics. Some professionals were concerned CM would shift focus from treatment needs to monitoring behaviours like urine samples [43]. Yet, these concerns often diminish once CM is implemented [37]. Clients who experienced CM found it validating and non‐judgmental and shifts the focus from punitive measures to positive reinforcement, which in turn can enhance trust between clients and clinicians and strengthen the therapeutic relationship [41]. The strength of the existing relationship might play a role in how CM is received, with some arguing that a poor relationship with their treatment provider would make the reinforcer less genuine, and therefore, change their interaction with the intervention [41]. Moreover, a strong existing relationship was considered vital for protecting against ‘drop‐out’ in the event of failure [35].

Another challenge is the potential manipulation of CM programmes. This raises concerns about fairness and programme integrity, particularly drawing attention to questions such as who exactly should be offered CM and for what behaviour. Poor treatment engagement and retention was flagged as a significant challenge within the substance use treatment sector. For some addiction specialists, using CM to encourage initial contact with services was considered a critical first step before important therapeutic work could begin. However, delivering CM in adjunct to constructive, recovery‐oriented activities that foster meaningful therapeutic interactions was deemed crucial in ensuring CM was not being used to reinforce surface‐level engagement [51].

To address ethical concerns, structured protocols and behaviour verification methods (e.g. urine tests, biometrics) are recommended to ensure integrity and reduce manipulation [63]. Organisational backing, thorough training, endorsement and CM advocates is critical to broader professional acceptance [64]. CM gains greater support when clearly presented as a structured, evidence‐based component of care rather than a standalone approach [65].

### Macro‐level (political and systemic challenges)

At the macro level, a key facilitator to CM implementation is its recommendation in the clinical guidelines. Despite this, CM is often hindered by political and systemic constraints. One of the primary barriers is the limited availability of NHS and third‐sector resources. Healthcare systems, particularly in the United Kingdom, face competing priorities and CM is often deprioritised because of budgetary constraints [51]. General disinvestment in local authorities and public health is argued to be responsible for hindering the adoption of not only CM, but also other evidence‐based interventions. The direct costs associated with CM, including staff training and incentives can be a deterrent for policymakers who prefer lower‐cost interventions. However, arguments about costs involved seem to be bound up with more complex views on the ethics of CM and resource distribution. Clinicians argued that perceived cost needs to be addressed at a higher organisational and treatment system level [49].

Studies exploring the acceptability of CM have highlighted professionals' perspectives on how political sensitivities and negative media portrayals contribute to resistance against CM [43, 49, 51]. Although not necessarily endorsing such objections, professionals highlight how some critics might argue that providing incentives, particularly financial, are an inappropriate use of public funds. Public perception plays a crucial role in shaping policy, and misrepresentations of CM in the media can contribute to stigma and reluctance to adopt it on a larger scale. In one study, professionals argued that societal and media perceptions of CM and portrayal of it as a bribe, rather than a legitimate therapeutic tool, are responsible for the lack of adoption [51].

There is also ongoing debate about CM being incompatible with harm minimisation approaches, which are central to many UK drug and alcohol treatment services. Some professionals argue that traditional CM protocols with their escalating incentives and resets for non‐attainment of the target behaviour aim to prevent relapse rather than prioritise harm reduction [43]. Although the evidence base consistently endorses the use of CM to promote abstinence from nicotine, alcohol and illicit substances, CM interventions targeting attendance, medication adherence and vaccination uptake have also yielded medium‐ to large‐sized effects [32, 66, 67, 68]. That being said, substance use behaviours consistent with harm reduction agendas, such as reduction in substance use, are more complex because of the difficulty in objectively detecting this behaviour [51]. Future research needs to consider how CM protocols can be adapted to accommodate reductions in substance use, such as using tests that can detect reductions in use or minimising the impact of incentive resetting.

Despite these challenges, several factors can drive CM adoption at the macro level. A strong evidence base supports the effectiveness of CM, with numerous RCTs demonstrating its efficacy in promoting abstinence and treatment adherence. National guidelines, including the UK's NICE [15] and the ‘Orange Book’ [16], already recommend CM as a treatment option for SUD, however, expanding awareness of these guidelines among policymakers and clinicians could help facilitate broader adoption. Finally, the development of an implementation strategy for CM can address many of the barriers at the micro, meso and macro level. This includes clear funding mechanisms, integration into clinical pathways and policy advocacy to counter misinformation.

### Limitations

A key limitation of this scoping review is the inclusion of studies addressing a wide range of behaviours, such as abstinence, vaccination adherence and problem gambling, which differ substantially in their underlying mechanisms and intervention contexts. This heterogeneity restricts direct comparison of effectiveness, so findings should be interpreted with caution. Furthermore, by design, this scoping review set out to capture all peer‐reviewed published studies evaluating the effectiveness, feasibility and acceptability of UK‐based CM. As such, the review was not limited to controlled trials. The review included two non‐controlled retrospective cohort design studies [30, 31]. Although these studies are valuable for understanding the impact of incentive‐based programmes within real‐world clinical settings, one should be cautious when inferring causality for several reasons, including a lack of control condition and a multi‐component design, making it difficult to attribute outcomes solely to the intervention. Regarding the latter point, the complexity of the intervention evaluated in one included RCT could make it more difficult to isolate which components were responsible for the outcomes reported, such as the CM intervention or another psychosocial intervention delivered in adjunct [29].

Furthermore, although several studies were delivered within real‐world clinical settings, thereby increasing their ecological validity, variation in implementation fidelity could have negatively impacted the effectiveness of these interventions. Although implementation efforts must ensure interventions are integrated in a sustainable way that fits current practice, doing so within resource‐limited NHS and third‐sector settings quite often results in a ‘diluted’ version of CM being delivered. High frequency behaviours (e.g. substance use or medication consumption) should be monitored as often as possible to establish a strong contingency between the target behaviour and reinforcer. Diluting CM by infrequent monitoring of the target behaviour or removing structure undermines its behavioural effectiveness [14]. Likewise, immediacy is important, and reinforcers that are delayed in delivery are less valuable than those provided immediately [13, 14, 69, 70]. Although such adaptations may increase political and ethical acceptability, they often lead to weaker or inconsistent outcomes. The implications of such are discussed more widely elsewhere [71].

Infrequent monitoring of the target behaviour is not the only key issue potentially impacting CM fidelity in the included studies. The use of low‐value consequences in incentive‐based programmes risks undermining the core principles of CM and reducing its ability to encourage significant behaviour change. A key principle of CM is the use of reinforcers that are meaningful and of sufficient value, motivating a person to repeat and sustain the behaviour. When low‐value reinforcers or non‐reinforcing outcomes are used, the effectiveness of CM can be significantly compromised [13, 72]. Similarly, behaviour change is more likely to occur when the value of the reinforcer escalates over time [73, 74]. Although low‐value non‐escalating reinforcers might be more ethically and pragmatically attractive in low‐resource settings or where the use of CM is viewed as controversial, client engagement is potentially undermined if the perceived value is seen as inadequate or tokenistic. To optimise CM's impact, reinforcers offered must be meaningful, appropriately scaled and delivered consistently and contingently on exhibition of the target behaviour.

## CONCLUSION

Although CM has demonstrated considerable promise as an evidence‐based intervention for SUD and related behaviours, its successful integration within UK services will require sustained commitment at multiple levels. The implementation of CM in UK substance use treatment is shaped by a complex interplay of micro, meso and macro level factors, as outlined in this review. Future work should focus on adapting CM protocols to local contexts, evaluating long‐term outcomes and establishing supportive infrastructure, including policy, training and funding, to enable effective and equitable implementation in real‐world settings. Addressing the structural and attitudinal barriers identified in this review will be critical for realising CM's full potential within the UK addiction treatment landscape.

### Key recommendations for research, policy and practice

- Ensure fidelity by adhering to CM core principles: maintain immediacy, consistency and meaningful reinforcer value.
- Align CM with harm reduction and UK treatment philosophies: adapt protocols to support not just abstinence but also reductions in use, treatment engagement and health‐promoting behaviours.
- Invest in training, technology and implementation support: provide workforce education, digital tools and administrative infrastructure to reduce the burden and enhance delivery fidelity.
- Embed CM in existing frameworks: integrate CM into policy and funding frameworks to normalise its use through commissioning. Support its delivery in adjunct to evidence‐based treatment approaches, and counter stigma with public and professional advocacy.
- Strengthen research on CM implementation: assess sustainability, cost‐effectiveness and integration within clinical settings.
- Use theories and frameworks from the field of implementation science to improve the adoption of CM in the United Kingdom.

## AUTHOR CONTRIBUTIONS

**Carol‐Ann Getty:** Conceptualization (lead); data curation (equal); formal analysis (equal); methodology (lead); project administration (lead); visualization (lead); writing—original draft (lead); writing—review and editing (lead). **Tricia McQuarrie:** Data curation (equal); formal analysis (equal); writing—review and editing (supporting). **Eileen Brobbin:** Data curation (equal); formal analysis (equal); writing—review and editing (supporting).

## DECLARATION OF INTERESTS

C.A.G. is involved in research projects that, through sub‐contracts, use the capability of the app‐development company CMI, however, not related to this research.

## Data Availability

Data available on request from the authors.

## Footnotes

1 The term ‘successfully completed treatment’ refers to individuals who have achieved all the goals outlined in their care plan, overcome dependent use of the substances that brought them into treatment or ceased any pharmacological interventions. It aligns with the criteria used by the National Drug Treatment Monitoring System (NDTMS) to determine successful treatment completion.

2 Separate studies using the same sample are counted once.
